# Bacteria derived extracellular vesicles in the pathogenesis and treatment of gastrointestinal tumours

**DOI:** 10.3389/fonc.2022.1103446

**Published:** 2023-01-23

**Authors:** Yang Ge, Fengyuan Sun, Bo Zhao, Fanyang Kong, Zhaoshen Li, Xiangyu Kong

**Affiliations:** ^1^ Changhai Clinical Research Unit, Changhai Hospital, Naval Military Medical University, Shanghai, China; ^2^ Department of Gastroenterology, Changhai Hospital, Naval Military Medical University, Shanghai, China; ^3^ National Key Laboratory of Medical Immunology & Institute of Immunology, Second Military Medical University, Shanghai, China

**Keywords:** extracellular vesicles (EVs), cancer, immunotherapy, TLR, LPS, PAMP

## Abstract

Extracellular vesicles are fundamentally significant in the communication between cells. Outer Membrane Vesicles(OMVs) are a special kind of EVs produced by Gram-negative bacteria, which are minute exosome-like particles budding from the outer membrane, which have been found to play essential roles in diverse bacterial life events, including regulation of microbial interactions, pathogenesis promotion, stress responses and biofilm formation. Recently, and more researches have explored the substantial potentials of EVs as natural functional nanoparticles in the bioengineering applications in infectious diseases, cardiovascular diseases, autoimmune diseases and neurological diseases, such as antibacterial therapy, cancer drugs and immunoadjuvants, with several candidates in clinical trials showing promising efficacy. However, due to the poor understanding of sources, membrane structures and biogenesis mechanisms of EVs, progress in clinical applications still remains timid. In this review, we summarize the latest findings of EVs, especially in gastrointestinal tract tumours, to provide a comprehensive introduction of EVs in tumorigenesis and therapeutics.

## 1 Introduction

**Figure 1:**
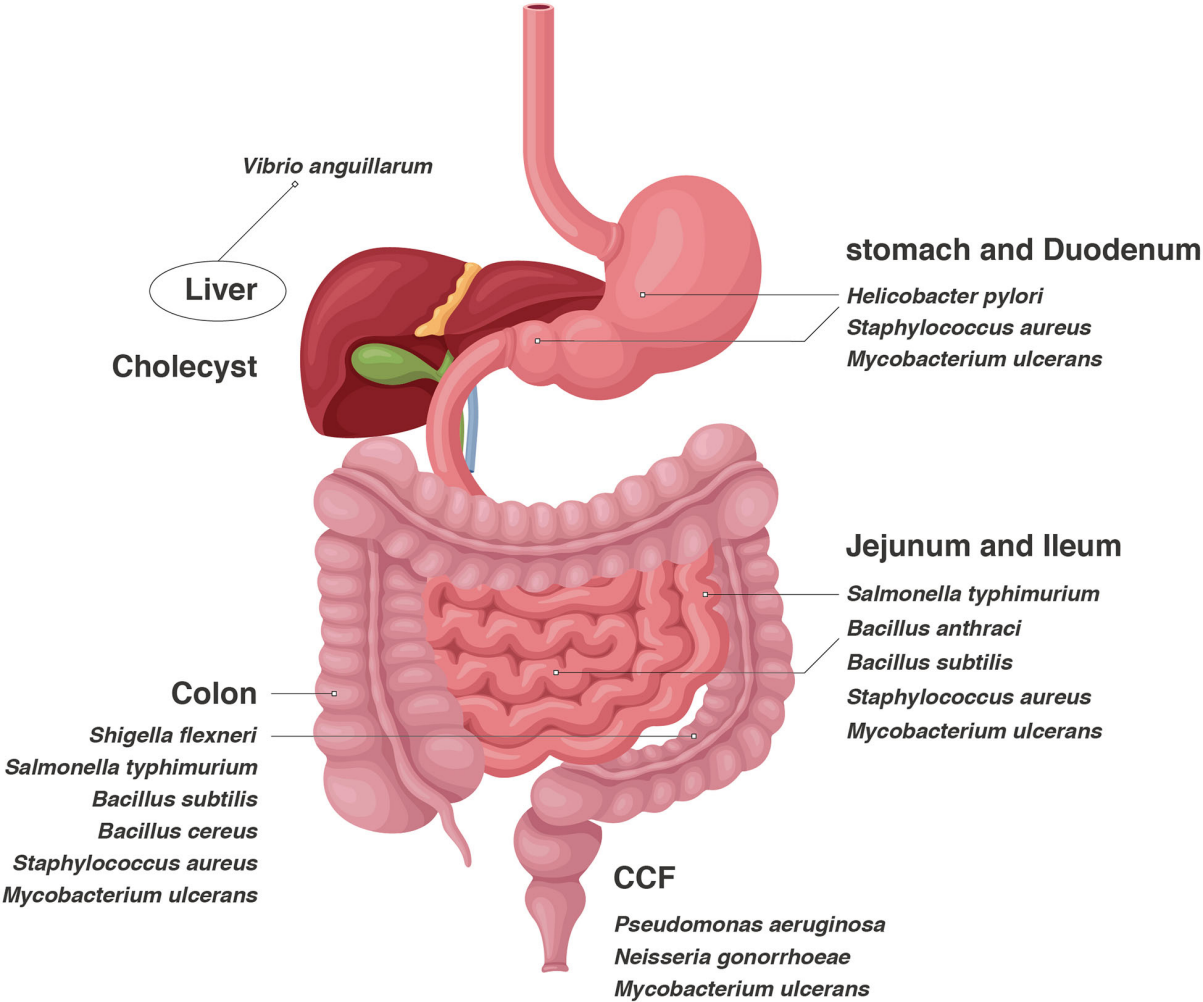
Bacteria are distributed in the whole digestive tract, and different bacteria tend to reside in specific organs.

During bacterial growth, extracellular vesicles(EVs)are released from the cell surface after vesiculation of the outer membrane(OM) (1–6). Bacterial EVs were first discovered in *Escherichia coli*. Since then, numerous Gram-negative bacteria have been found to produce and secrete EVs, such as *Pseudomonas aeruginosa*, *Salmonella typhimurium* and *Helicobacter pylori*. Additionally, Gram-positive bacteria such as *Bacillus anthracis, Bacillus subtilis* and *Staphylococcus aureus* can also produce EVs (7) (**Table 1** and **Figure 1**). EVs are spherical particles with an average diameter of 10–300 nm that are formed by budding vesicles originating from the OM that bulges outward and enwraps the surrounding cytoplasm, proteins, DNA and RNA (8). The EV proteome contains proteins not found in parent cells. A multifunctional vaccine platform based on bacterial EVs has received incremental attention in recent years (9). Immune responses can be triggered both innately and adaptively by EVs, which are intrinsically immunostimulatory (10). Moreover, their nanoscale size facilitates their processing by immune cells. EVs can be modified in several ways, such as *via* genetic manipulation, vector charging and nanoparticle wrapping, to create multifunction platforms that can be used to treat diverse diseases (11). EVs have emerged as useful components for formulating new vaccines. Additionally, EVs can retain and distribute nutrients, virulence factors and toxins while also using host-produced antimicrobial molecules (12). These natural nanostructures have many useful biological functions for immune-linked inhibition (13).

**Table 1:** Bacteria reported to be able to produce OMVs.

Bacteria Strain	Type of Gramstain	Reference
Escherichiacoli	G-	Knox KW, Vesk M, Work E, et all. Bacteriol, 1966, 92(3):1206-1217.
Pseudomonasaeruginosa	G-	Bauman SJ,Kuehn MJ,et al.Microbes Infect,2006,8(8):2400-2408.
Shigellaflexneri	G-	Kadurugamuwa JL,Beveridge TJ,et al.Microbiology,1999,145(Pt 8):2051-2060.
Salmonellatyphimurium	G-	Garcia-del Portillo F,Stein MA,Finlay BB,et al. Infect Immun, 1997,65( 2) : 24-34.
Helicobacterpylori	G-	Fiocca R,Necchi V,Sommi P,et al. Pathol,1999,188(5):220-226.
Borreliaburgdorferi	G-	Shoberg RJ, Thomas DD, et al. Infect Immun,1993,61(2):3892-3900.
Vibrioanguillarum	G-	Hong GE,Kim DG,Park EM, et al.Biosci Biotechnol Biochem,2009 73(8):437-439.
Neisseriagonorrhoeae	G+	Pettit RK, Judd RC, et al.Mol Microbiol,1992,6(10):729-734.
Bacillusanthraci	G+	Dorward DW,Garon CF,et al. Appl Environ Microbiol,1990,56(9:1960-1962.
Bacillussubtilis	G+	Dorward DW,Garon CF,et al. Appl Environ Microbiol,1990,56(9:1960-1962.
Bacilluscereus	G+	Lee EY,Choi DY, Kim DK, et al. Proteomics,2009,9(3):5425-5436.
Staphylococcusaureus	G+	Rivera J,Cordero RJ,Nakouzi As,et al. Proc Natl Acad Sci USA,2010,107(11):19002-19007.
Mycobacteriumulcerans	G+	Rivera J,Cordero RJ,Nakouzi As,et al. Proc Natl Acad Sci USA,2010,107(11):19002-19007.

## 2 Composition and structure of EVs

Pathogenic and non-pathogenic Gram-negative and -positive bacteria release nanosized granules of 20–300 nm in diameter from their OM into the extracellular environment (14). Bacterial EVs play a major role in a wide range of biological processes, including virulence, aclinic gene output, cellular metabolism, bacteriophage infection and cell-to-cell interactions (15). EVs are usually generated *via* controlled blistering of bacteria. Given the core components and capabilities of EVs, they can be used to treat gastrointestinal neoplasms (16).

### 2.1 Proteins

**Figure 2:**
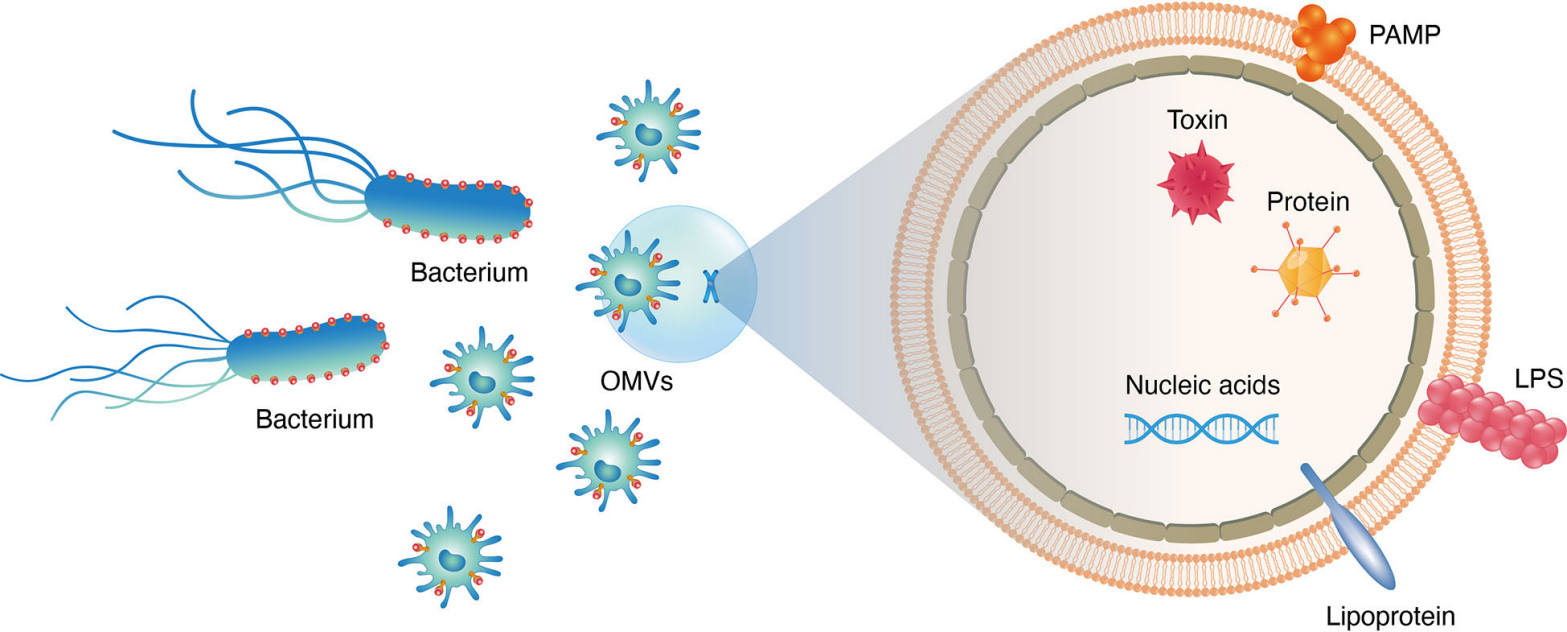
Different functional constituents existed in bacteria derived OMVs, e.g., proteins, nucleic acids, lipids, virulence factors and PAMPs.

Abundant OM proteins(OMPs; i.e., OmpA, OmpC and OmpF), membrane interstitial proteins(AcrA and alkaline phosphatase), misfolded proteins and virulence factors associated with adhesion and invasion of host organs are found in EVs (17) (**Figure 2**). More than 3500 proteins belonging to various functional classes have been characterised using MS-based proteomic approaches after purification of EVs *via* ultracentrifugation. According to a global proteomic analysis, EVs lack components of the inner cell membrane and cytoplasm, implying that vesicle formation is a directed process instead of a random process (18). EVs are formed *via* blistering and swelling and mostly consist of interstitial proteins and OM protein envelope components. Over the past few years, high-throughput proteomic data of EVs collected from pathogens and commensal bacteria have revealed new functional proteins that can be used for biomedical applications (19). Therefore, the integration of proteomic data under various physiological conditions may facilitate the development of EV-based biomimetic materials (20).

### 2.2 Nucleic acids

Bacterial EVs carry lumen- and surface-associated DNA, RNA, purified plasmids, bacteriophage DNA and chromosomal DNA(**Figure 2**). A study revealed the presence of DNA in EVs by treating bacterial samples with DNase (21). Resistance to luminal DNA was maintained even after finishing the course of treatment (22). Additionally, a few different forms of *Escherichia coli(E. coli), Neisseria gonorrhoeae(N. gonorrhoeae), Pseudomonas aeruginosa(P. aeruginosa)and Haemophilus influenzae(H. influenzae)*have been reported (22). Genomic DNA has been found in EVs released by the commensal bacterium *Enterobacter cloacae* (18). Over the past few years, small noncoding RNAs(sRNAs)have been discovered in bacterial EVs (23). These sRNAs have a length of 50–400 nucleotides (24). For instance, Ghosal et al. reported that sRNAs in *E. coli* MG1655-derived EVs had a length of 15–40 nucleotides and performed numerous functions (25, 26). To escape degradation by RNases and perform biological functions in the related host, bacteria may carry functional sRNAs in EVs. Although functional sRNAs native to bacterial EVs have been identified in various studies *via* high-throughput RNA sequencing (26), their function remains unclear.

### 2.3 Lipids

Lipids are one of the key components of the structure of bacterial EVs. Based on the enriched and excluded EV components, some lipid components may be important for EV biogenesis. In Gram-negative bacteria, lipopolysaccharides(LPSs)and phospholipids(PLs)are the most common lipid components (27) (**Figure 2**). PLs constitute the inner layer of the OM, whereas LPSs are found only on the outer surface. The content of PLs varies among Gram-negative bacteria. Phosphatidylethanolamine(PE), phosphatidylglycerol(PG)and lysophosphate(LY)are the main PLs in *E. coli* EVs, whereas PG, PE and stearic acid are the major lipid components in other bacterial EVs (28). The three major classes of OM lipids in the myxobacterium *Sorangium cellulosum* include sphingolipids(SLs), which have long-chain amino alcohol backbone; glyceryl ether lipids(GELs)and bird-containing lipids amino acid lipid (OLs). In group A *Streptococcus* (GAS) -derived EVs, more than 85 glycerolipids (GLs)are present, including anionic and cationic PLs (29). In Hp-derived EVs, cardiolipin(CL)is a major lipid component. In Gram-negative bacteria, the OM contains both neutral (O-LPS)and negatively charged (A) -LPSO antigenic residues. EVs derived from Gram-negative bacteria contain only A-LPS (17). Gingivalase, a protein highly abundant in *P. gingivalis*, is eliminated in the A-LPS mutant strain, which highlights the importance of EV packaging. A recent study demonstrated that delivery of *P. gingivalis* SL *via* EVs can alter host inflammatory responses. Because EVs derived from *Singomonas paucimobilis* contain SL, a lipid class that plays an important structural and signalling role in eukaryotes, this bacterium may be used to build a system in which host–bacteria interactions are exploited.

### 2.4 Virulence factors

EVs can directly communicate between bacteria and the host by delivering toxins and enhancing bacterial virulence in host cells to benefit pathogenic bacteria (30) (**Figure 2**). As an example, EstA, a bacterial virulence factor derived from the EVs of *P. aeruginosa*, induces nitric oxide and pro-inflammatory cytokines during macrophage interaction with host cells (18). As a result of these virulence factors, host cells are dysregulated by proteins released by EVs, thereby affecting proteolysis, ion transport and ion binding (18). EVs derived from *H. pylori* exhibit Lewis antigens on their surface and can activate the immune system of the host. As a result, EVs directly bind to anti-Lewis antibodies in serum, thereby decreasing the self-defence ability of host cells (31). EVs play an important role in a wide range of biomedical applications because they can deliver various toxins and activate the defence system of the host. Therefore, EVs can be used to develop nano-platforms.

### 2.5 PAMPs on EVs

LPS is the most potent and immunostimulatory component of EVs (32). EV-based vaccines with diametrically opposed views were developed by two scientists who discovered LPS. Owing to the risk of inducing systemic reactogenicity when administered to humans(which is why LPS was historically called an endotoxin), LPS poses safety concerns. However, the presence of LPS supports the ability of EVs to stimulate the immune system effectively. Developing EV-based vaccines requires maintaining the balance between reactogenic risk and the ability to stimulate an effective immune response (33).

The lipid A subunit of LPS directly interacts with toll-like receptor (TLR) 4 (32), and changes in its composition significantly affect its binding and recognition, resulting in decreased agonistic or antagonistic effects, dimerisation of TLR4/MD-2 and downstream signalling.

The basic lipid A structure in Gram-negative bacteria is phosphorylated at positions 1 and 40 by a glucosamine disaccharide and Acylated composition at positions 2 and 3 with R-3-hydroxymyristate at positions 20 and 30 (known as lipid IV A). Several enzymes decorate this structure at various positions, especially fatty acids of varying length and saturation, or alter it by adding (or removing)phosphate groups of glucosamine (34). Bacteria use changes in the basic structure of lipid A to respond to various shocks and modulate host–pathogen interactions, thereby evading innate immune detection (34, 35). In *E. coli* and *Shigella*, the late acyltransferases HtrB (36)(also known as LpxL) and MsbB (also known as LpxM)transfer lauroyl and myristoyl fatty acids to positions 30 and 20, respectively, resulting in the highest reactivity of lipid A (phosphorylated at positions 1 and 40, acyl chain length of 12–14 carbons and asymmetric distribution) (37). PagP (38) catalyses the addition of a seventh fatty acid chain to hexaacylated lipid A *via* 2-O-palmitoylation, whereas LpxR (39) or PagL (40) catalyses deacylation. EptA and ArnT add phosphatidylethanolamine or arabinose to 1 and 40 phosphate groups (41), respectively, whereas LpxE degrades one phosphate group. Hydroxylation of LpxO is another widely described example of an enzymatic reaction that targets different lipid A sites (42, 43). Additionally, different enzymes can act on the same site of lipid A molecules, catalysing the substitution of some acyl chains by other amino chains(LpxP can add palm oleyl chains to the same site as HtrB) (44). Depending on the substrate specificity, enzymes have different acyl chains(acyl chains of varying lengths of 2–4 carbons are attached depending on stress conditions or the metabolites present). Therefore, the various types of lipid A provide bacteria with different abilities to activate TLR4, and hence, EVs have different abilities to activate TLR4, with different structures having different agonistic and antagonistic abilities (45).

A majority of genes involved in lipid A biosynthesis are essential for bacterial membrane integrity. Therefore, to alter the acylation state of lipid A with minimal defects in bacterial growth, genes encoding late acyltransferases should be inactivated individually or in combination. For instance, HtrB or MsbB deletion results in the production of EVs with predominantly pentaacylated lipid A, with EVs carrying wild-type lipid A for each. However, these EVs significantly decrease the levels of proinflammatory cytokines. In *Salmonella enterica* and *Francisella tularensis* (33, 46, 47), dephosphorylation of lipid A has been examined preclinically by knocking down the enzyme encoded by lpxE or by overexpressing the deacylase encoded by lpxR, which resulted in the cleavage of two fatty acid chains or PagL, which is a 3-O-degradation enzyme. For the development of meningococcal vaccines based on EVs, the endotoxin-reducing activity of lipid A is also essential. The LPS content of EVs is reduced when a detergent-based extraction method(for example, Bexsero)is used. In addition, genetically detoxified LPS can be used to generate meningococcal strains, specifically by knocking out the L1 (48) and L2 (49) genes. These genes produce pentaacylated lipid A, which significantly attenuates endotoxin activity, both preclinically and clinically.

**Figure 3:**
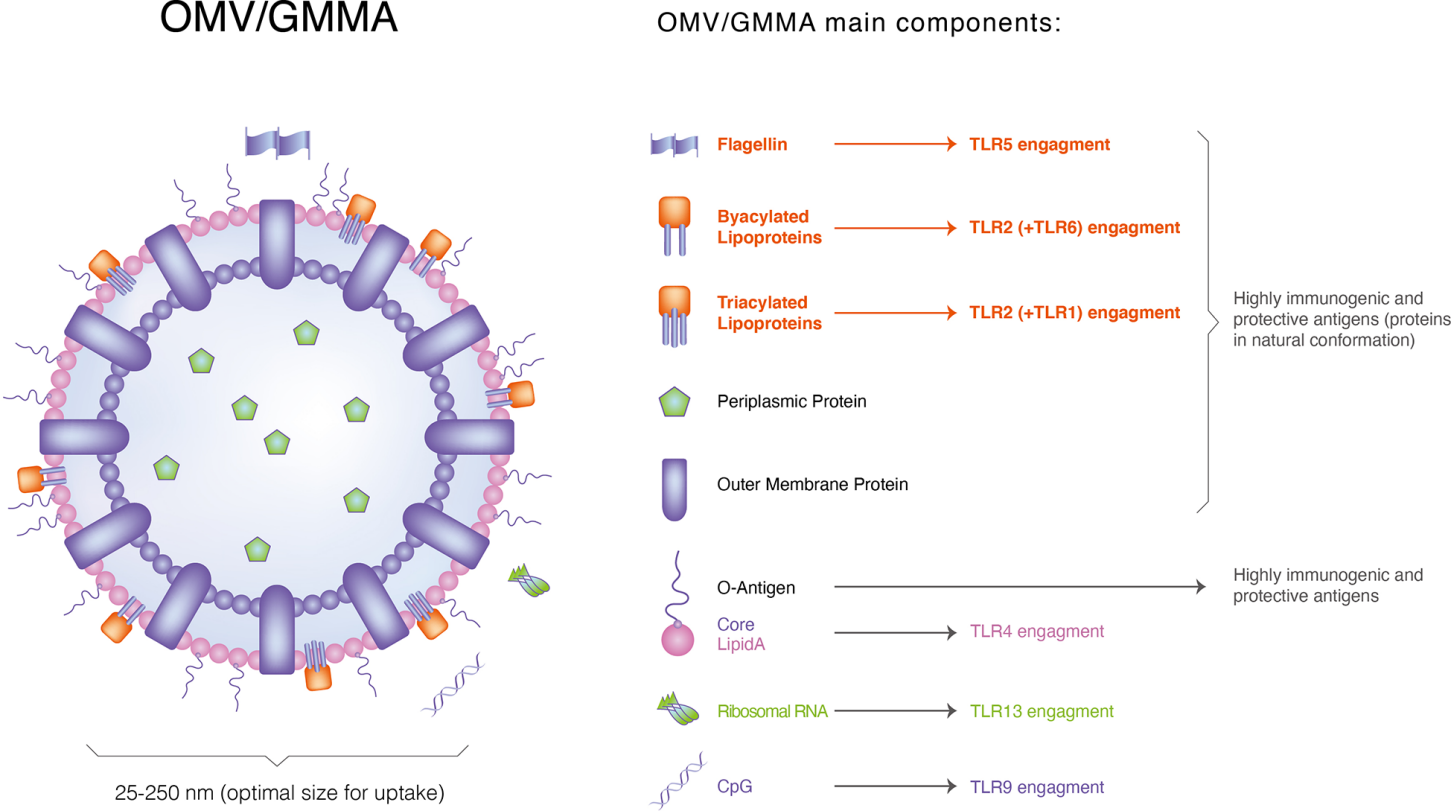
Those highly immunogenic components that exist in OMVs.

Other PAMPs have also been found in EVs. Stably transfected human embryonic kidney cells expressing only specific TLRs consistent with their native lipoprotein content have been shown to efficiently activate TLR2 *via* GMMA in *Shigella* (46), *Salmonella* (50) and *Neisseria meningitidis*. Additionally, targeted blockade of TLRs in human PBMCs, alone or in combination, elucidates the relative contribution of TLR2, TLR4 and TLR5 to the ability of *Shigella* and *Salmonella* GMMA with modified lipid A to induce pro-inflammatory responses (**Figure 2**). The contribution of TLR4 to pro-inflammatory responses is significantly reduced to a level where TLR2 activation mainly mediates the response, whereas the relative contribution of PRRs other than TLR2, TLR4 and TLR5 is negligible(<10%). Non-LPS PAMPs can enhance immune responses to vesicles (51) in LPS-deficient EVs in *Neisseria*. Flagellin and CpG DNA have been found in *Salmonella* GMMA and *Pseudomonas aeruginosa*-derived vesicles (52). However, whether they directly affect the response of the host to intact vesicles containing LPS remains unclear. *Shigella* GMMA preparations, possibly purified with GMMA, have low levels of cytoplasmic proteins(mainly ribosomal proteins) (53) (**Figure 3**).

## 3 EVs in bacteria-host crosstalks

Cellular components and nanoparticles can be delivered *via* EVs. These specific cargoes facilitate bacterial communication, immunomodulatory host–bacteria communication and cell detoxification.

### 3.1 Bacteria–bacteria communication

EVs can interact with other microorganisms and hosts in the environment and have specialised communication systems under variable or challenging environmental conditions, including quorum sensing(QS), biofilm formation, nutrient acquisition, antibiotic resistance and competition with or defence against other microorganisms (54). *Paracoccus denitrificans*- and *Vibrio harveii*-derived EVs use quorum sensing under activated quinolone signalling(PQS)to interact with other bacteria and host cells, such as C16-HSL and CAI-1 (54). In bacterial communities, EVs share biofilm substrates to improve their ability to absorb nutrients and survive. Plankton- and biofilm-derived EVs in *P. aeruginosa* differ in quantity, quality, proteolytic activity and antibiotic-binding ability and participate in some activities of biofilms (55). Additionally, EVs derived from antibiotic-resistant strains have been shown to transfer antibiotic resistance genes and proteins to susceptible strains (9). For example, *Acinetobacter baumannii*-derived EVs contain carbapenem-resistance-related genes that can be horizontally transferred to carbapenem-resistant organisms (56). Furthermore, *E. coli* produces EVs that contain colistin, which degrades antimicrobial peptides such as melittins (57). The EVs of *Moraxella catarrhalis* and *Staphylococcus aureus* carry beta-lactamase, which allows them to escape antibiotics.

**Figure 4:**
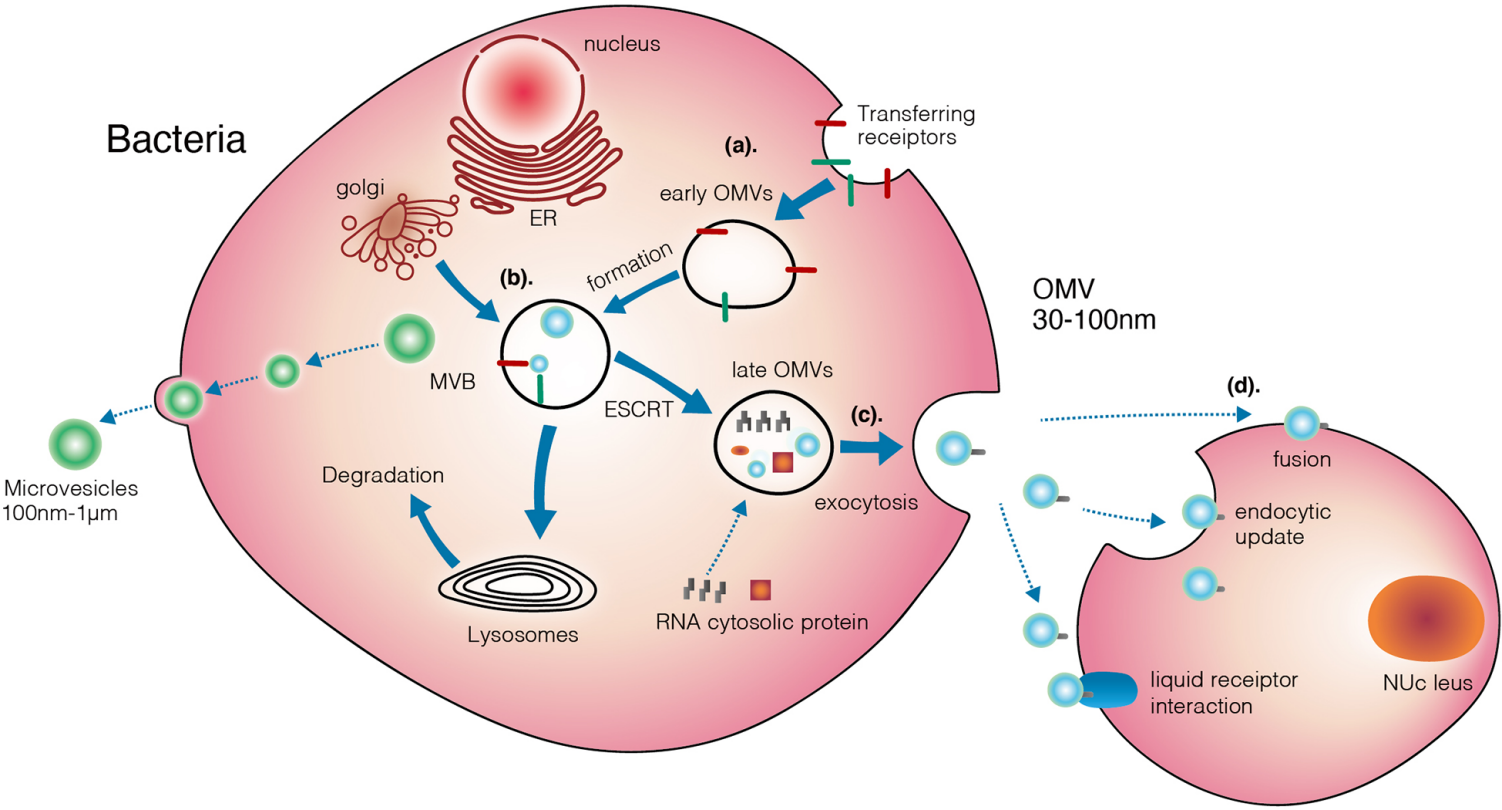
Three different mechanisms that mediating transferring of OMVs from bacteria to target cells.

Horizontal gene transfer is a primary EV-mediated mechanism. However, only a few studies have examined the gene transfer ability of EVs. There are three steps in the process as follows: DNA is released from EV-encapsulated donor bacteria, EVs are incorporated with recipient bacteria and genetic material is transferred into the cytoplasm of the recipient bacteria. Several instances and related factors have been reported;however, this review does not address them in detail. Further investigation is required to understand the mechanisms underlying the loading of EVs and their attachment to recipient bacteria. In addition, novel mechanisms underlying EV-mediated bacterial communication should be investigated in species with varying lipid compositions (58) (**Figure 4**).

### 3.2 Immunomodulatory host–bacteria communication

Because EVs contain many immune-reactive proteins originating from their parent bacterium, they modulate innate immune responses in the host without direct host cell–bacteria interactions (59). In terms of structure and biological activity, bacteria-derived EVs are similar to those secreted by mammalian cells. EVs are required to alter the integrity of the epithelial barrier to maintain homeostasis in mammalian cells associated with inflammatory and metabolic diseases and induce immunomodulatory activity (60). Furthermore, the gut microbiome and intestinal epithelium interact to maintain barrier integrity. For example, EVs derived from probiotic *E. coli* and commensal *ECOR63* can enhance barrier function (61).

In addition to interacting with host epithelial cells, in which EVs derived from both Gram-negative and -positive bacteria are involved, EVs directly affect immune cells, either by activating or suppressing immune responses. The gastric mucosa of patients with *H. pyroli* infection has been reported to contain EVs (62). *S. typhimurium*-derived EVs promote flagellin-mediated caspase-1 activation and IL-1β secretion in an endocytosis-dependent manner (59). EVs derived from *H. pyroli*, *Neisseria*, *Pseudomonas*, *Campylobacter* and *Vibrio* induce the secretion of interleukin-8(IL-8), a chemokine produced by tissues and blood cells, in non-phagocytic epithelial cells (63). EVs derived from Gram-positive bacteria such as *Lactobacillus plantarum* and *L. sakei* can protect against pathogen infection by stimulating host defence-related genes. The TLR8 and NF-κB signalling pathways in *A. actinomycetemcomitans*-derived EVs can activate the pro-inflammatory cytokine TNF-α (64).

### 3.3 Cell detoxification: Toxins, adhesions and virulence factors

EVs derived from many pathogenic bacteria contain toxins, degrading enzymes, virulence factors and pro-inflammatory compounds. These components can be used as tools to facilitate the contact of EVs with host cells. The Apx toxin (Shiga toxin)is activated by *A. pleuropneumoniae*-derived EVs and induces haemolysis and cytolysis. *Salmonella typhimurium*, however, possesses a special secretion system called the type III secretion system (T3SS)that secretes more than 40 virulence factors (65). EV-related virulence factors are not completely understood;however, it is evident that the survival of *Salmonella* is reduced without these factors. Although these toxins are found on the outer surface of vesicles, they can often resist proteases (65).

Gram-negative and -positive bacteria secrete EVs with particle sizes of 20–300 nm. EVs contain biologically active proteins, toxins, virulence factors and immunogenic materials that stimulate bacteria–host interactions, through which vaccines are developed (65). The non-replicative nature of EVs makes them relatively safe, and their biological roles are unpredictable under different environmental stresses. Therefore, *in vivo* biomedical applications of EVs can be more effective.

## 4 The role of bacteria EVs in the pathogenesis of certain gastrointestinal cancers

### 4.1 Pancreatic Ductal Adenocarcinoma (PDAC)

The human microbiome is an inherent element in the lifelong conservation of fitness and immune system homeostasis. It plays a fundamental role in tumorigenesis, cancer course and disease feedback. Approximately 85% of cases are related to PDAC, with only 24% of patients surviving 1 year after diagnosis, and the 5-year survival rate is approximately 5–7%.

*Porphyromonas gingivalis* and *Aggregatibacter actinomycetemcomitans* have been associated with an increased risk of PDAC (66). *Porphyromonas gingivalis* and *Actinobacillus congeners* were found to increase the risk of PDAC by 59% and 50%, respectively, in a nested case–control study that involved 361 patients with PDAC and 371 control patients and spanned over a decade (67). Periodontitis plays an important role in the pathogenesis of PC, and the two abovementioned oral microbes are causative agents (66, 68). Microbes from the mouth can migrate and translocate to the pancreas and other organs through the vascular network and/or the digestive tract. When located at a distance, disruption of the immune system and inflammation can accelerate cancer progression (69).

*Porphyromonas gingivalis* is a major pathogen associated with oral and gastrointestinal cancers (70). It exerts its toxicity through various virulence factors;however, recent studies have revealed a unique protein called PPAD. Gabarrini et al. reported that PPAD homologues are found in other *Porphyromonas* species, such as *P. gulae* and *P. loveae (* 71, 72 *)*. Several studies have suggested that PPAD plays a role in the tumorigenesis of PC through KRAS mutation and P53 activation. Normally, the tumour suppressor gene P53 causes cell cycle arrest, thus allowing DNA repair in damaged cells (73). If the damage cannot be repaired, the cells become apoptotic targets. Approximately 50% of human cancers have mutations in p53 (74). The KRAS gene is an oncogene that usually has GTPase activity, and mutations in this gene interfere with its activity and may lead to inappropriate cell proliferation and transformation, which are associated with tumorigenesis and a poor prognosis. KRAS mutations are associated with several pathological conditions with an aggressive phenotype and a poor prognosis. PDAC is usually associated with KRAS mutations. Studies on *Porphyromonas gingivalis* and PPAD have reported that rheumatoid arthritis is associated with both periodontitis and citrullination (75). PPAD can catalyse citrullination, a post-translational modification.

### 4.2 CRC

Chronic inflammation and changes in the diversity and function of the gut microbiota are accompanied by changes in immune responses. A major risk factor for colorectal cancer (CRC)is chronic inflammation. TLRs play an important role in inflammatory responses. They can sense microbial constituents, including nucleic acids, LPSs, peptidoglycans and bacterial EVs. EVs can carry or be decorated with these TLR activators. These microbial factors can boost tolerance or activate signalling pathways that result in chronic inflammation. In a survey, we discussed the role of the gut microbiome and EVs derived from gut bacteria in promoting the development of chronic inflammation and colitis-associated CRC. In addition, we discussed the role of TLRs (**Figure 3**) in mediating the microbiome inflammatory axis and subsequent tumour development.

#### 4.2.1 Factors inhibiting colorectal cancer

Acetate, propionate and butyrate are short-chain fatty acids (SCFAs)produced by intestinal bacteria (76). In the human colon, butyrate is mainly synthesised by glycolysis from hydrocarbons in Firmicutes spp., *Ruminococcus* spp. and sea squirrel families. The inhibitory effects of butyrate on cancer cell proliferation and cell death are attributed to its role in inhibiting histone deacetylase(HDAC) (77). Normal colon cells generate energy by oxidising butyrate through tricarboxylic acid oxidation (TCA cycle)or cytosolic acyl-CoA oxidation. Wahlberg et al. reported that cancer cells can alter their metabolic patterns even when oxygen is present and prefer the glycolytic pathway instead of the oxidative phosphorylation pathway(OXPHOS)to convert glucose into lactate. Glycolytic metabolism can be altered in cancer cells (78). Owing to the Warburg effect, colon cancer cells prefer glucose to butyrate for energy. Consequently, colon cancer cells accumulate high levels of butyrate, which inhibits HDAC (79). The levels of short-chain acyl-CoA dehydrogenase(SCAD), the primary enzyme catalysing mitochondrial butyrate oxidation, are decreased when butyrate directly enters the nucleus and inhibits histone deacetylase 1. As a result, autooxidation of butyrate in cancer cells is reduced, and butyrate accumulates in cancer cells, thereby inhibiting the development of cancer. Therefore, tumour cells are more sensitive to the inhibitors of HDAC than nontransformed ones (80).

Overexpression of microRNA-92a (miR-92a)promotes tumour growth and invasion in CRC by targeting KLF4 and downstream p21, whereas downregulation of miR-92a can result in the apoptosis of CRC cells (81). miR-92a can inhibit phosphatase and tensin homologue (PTEN) (82), which is an anti-oncogene found on chromosome 10q23 and is the most important negative regulator of PI3K signalling. Extracellular stimuli can activate PI3K, which converts phosphatidylinositol-4, 5-bisphosphate (PIP2) to phosphatidylinositol-3, 4, 5-trisphosphate (PIP3), which phosphorylates and activates the Akt pathway (83). To block PI3K signalling, PTEN dephosphorylates PIP3 and forms PIP2, thus blocking the PIP3K signalling cascade (84). Inhibition of miR-92a by butyrate can stimulate apoptosis and prevent the proliferation of colon cancer cells. Butyrate can also reduce the phosphorylation of Akt1 and ERK1/2 by blocking HDAC3 activity, thus inhibiting any subsequent cell movement and preventing the metastasis and invasion of CRC (85).

When miR-92a is overexpressed in CRCs, it targets KLF4 and downstream p21, promoting tumour growth and invasion. Similarly, reduced miR-92a expression can induce apoptosis in CRC. In response to extracellular stimuli (including insulin, growth factors and chemokines), PIP2 is converted to PIP3 by activated PI3K and Akt (protein kinase B)is phosphorylated and activated. Dose-dependent downregulation of miR-92a expression by butyrate *via* c-Myc may reduce the proliferation of colon cancer cells and stimulate apoptosis. In addition, butyrate inhibits the movement of cells by blocking the activity of HDAC3 and inhibiting the phosphorylation of Akt1 and ERK1/2, which eventually prevents tumour metastasis and invasion.

Tumour size and pathological stage (pTNM)are associated with decreased expression of miR-203 in CRC tissues and cells (86). Epithelial cells undergo epithelial–mesenchymal transition (EMT)during the early stages of cancer. During EMT, the loss of E-cadherin, an important component of adhesion, disrupts cell-to-cell contact. E-cadherin is degraded by Hakai, an E3 ubiquitin ligase that regulates cell adhesion by binding and degrading it. Based on the differentiated TNM staging(I–IV), Hakai expression is upregulated in colorectal adenocarcinoma and adenoma. Overexpression of Hakai in epithelial cells induces cellular transformation and mesenchymal and invasive phenotypes, whereas inhibition of E-cadherin promotes cell proliferation. miR-203 inhibits cell proliferation by directly targeting Hakai and reducing its levels. As part of the Crk-associated substrate (CAS)family, *NEDD9* is upregulated in multiple cancer types and participates in the adhesion and migration of cancer cells and tumour invasion. In CRC, *NEDD9* promotes EMT *via* the JNK pathway(c-Jun N-terminal kinase) (87). MiR-203 inhibits the proliferation and colonisation of CRC cells and tumour invasion and induces apoptosis by targeting *NEDD9*. Butyrate also upregulates miR-203, thereby inhibiting the proliferation and colonisation of CRC cells and tumour invasion and inducing apoptosis (88).

#### 4.2.2 Factors that promote colorectal cancer

Sulfate-reducing bacteria (SRB)metabolise sulfate and other sulfur-containing compounds, including taurine, in food, releasing hydrogen sulfide (H_2_S) (89). H_2_S promotes the growth of CRC cells. H_2_S levels are higher in the stool of patients with CRC than in the stool of healthy individuals. Additionally, faecal H_2_S levels are significantly higher among patients with colon tumours who have received sigmoid surgery than among healthy individuals of similar ages, suggesting that patients with colon cancer have a reduced ability to detoxify H_2_S (90). H_2_S may be involved in the pathogenesis of inflammatory bowel disease (IBD)and CRC. Several studies have reported the role of H_2_S in CRC. At physiological concentrations, H_2_S may promote cancer through inflammation and genotoxicity in addition to inhibiting butyrate oxidation and promoting cell proliferation *in vitro*. In the colon wall, where epithelial cells are exposed to bacteria and toxins, disulfide bonds are disrupted by H_2_S, leading to pro-inflammatory effects. A few studies have shown that H_2_S can prevent inflammation by protecting and rebuilding damaged mucous layers. H_2_S is produced by novel nonsteroidal anti-inflammatory drugs(NSAIDs). In several cancer cells, compounds that release H_2_S exhibit potent anticancer effects by inhibiting proliferation and/or inducing apoptosis, including CRC (91). The underlying mechanisms remain unclear but may be related to the inhibition of nuclear factor-κB (NF-κB)signalling by H_2_S, with an increase in intracellular calcium concentration as the secondary result. Overall, the role of H_2_S in CRC is controversial and warrants further investigation.

As a key virulence factor found in *Fusobacterium nucleatum*(Fn), FadA regulates cell adhesion and tumour invasion (92). Comparing specimens from CRC patients with normal tissues, FadA gene expression was significantly increased. pre-FadA has 129 amino acid (AA)residues, which are not secreted, whereas the mature FadA has 111 AA residues (93). As a result of intrinsic FadA and mFadA pre-complexes, Fn can bind to and invade host epithelial cells, which is important for Fn adhesion and cell invasion and results in detachment of CDH5 from cells, thereby increasing endothelial permeability and allowing bacteria to pass through vogue junctions, that FadA binds to host endothelial receptors and vascular endothelial globulin (CDH5) (92). In addition to binding to E-cadherin *in vitro*, FadA can bind to HEK293 cells, which are not cancerous, and CRC cells other than RKO cells. The E-cadherin receptor participates in Fn adhesion and CRC invasion through FadA. FadAc binds to E-cadherin and inhibits its tumour suppressor activity by phosphorylating and internalising it. As a result, β-catenin regulates the transcription and gene expression of NF-κB and Wnt, promoting the proliferation of CRC cells (94).

Intestinal bacteria such as *E. coli* and other Gram-negative bacteria produce colistin and cytolethal distending toxins(CDTs), which directly damage DNA in the large intestine (95–97). Biosynthetic processes produce colibactin, a heterogeneous ketone compound and non-ribosomal peptide complex. Certain strains that produce myxomycetin are associated. Eukaryotic cells can become senescent when colibactin damages double-stranded DNA and affects chromosome stability. Bacteria such as *E. coli* can modify the tumour microenvironment by secreting growth factors (98), leading to cellular ageing. The CDT family of toxins is produced by a wide range of Gram-negative bacteria, including *E. coli*, Actinomycetes, *Shigella* and *Helicobacter*. CDT has three subunits, namely, CdtA, CdtB and CdtC. CdtB is similar to DNAase I in that it causes damage to host DNA (99). In addition to mediating the interaction of toxins with cytoplasmic membranes, the CdtA and CdtC subunits are essential for internalising CdtB, which is part of an essential active subunit. CDT can cause DNA damage, leading to cell cycle arrest and cellular senescence. The presence of CDT plays a critical role in reducing the carcinogenic effects of *Campylobacter jejuni*. CDTs produced by *Campylobacter jejuni* damage host cell DNA and promote colorectal tumorigenesis by triggering cell growth and enhancing the nuclear translocation of β-catenin (99).

## 5 Bacteria EVs in the prevention of tumors

EVs activate inflammatory responses by activating TLRs (100) (**Figure 3**). TLRs sense microbial components, such as nucleic acids, LPSs, peptidoglycans and EVs. These TLRs can be decorated on EVs or carried as transmitters by them. As a result of these microbial factors, tolerance may be promoted or signalling pathways leading to chronic inflammation may be activated (100).

Hepatitis A, B and C viral infections cause the majority of hepatitis-related deaths, with HBV infection being the most common (101). Although there is an approved vaccine for HBV infection, it remains unknown whether HCV can be prevented *via* vaccination (102). HCV infection can be effectively treated with direct-acting antiviral therapy;however, prevention remains the most important challenge (102). In a study, *Lactococcus lactis* was engineered to produce polyhydroxybutyrate inclusions displaying the core antigen of the hepatitis C virus. Mice that received antigen-presenting particles produced more IFN-c, IL-17A, tumour necrosis factor-alpha and IL-6 and fewer IgG2c antibodies. Administration of Emulsigen with the vaccine produced strong IgG1 antibodies and cytokine responses. Vaccines can be developed against HCV infection using a fusion protein that encodes a truncated form of the core antigen fused with the nonstructural protein 3 (103). Subcutaneous administration of the antigen in combination with purified *N. meningitidis*-derived EVs in mice can increase the production of IgG antibodies, pro-inflammatory cytokines and granzyme B, suggesting that the combination may be used for developing vaccines against HCV infection (103).

## 6 Bacterial EVs and immunotherapy

### 6.1 EV in Immunotherapy

To interact with microbes and maintain a symbiotic relationship, the host requires a well-developed communication system with its symbionts and pathogens. Factors secreted by cells can be degraded by enzymes (e. g. proteases and nucleases). Prokaryotes and eukaryotes package cargoes (e. g. proteins, ribonucleotides and metabolites)in extracellular vesicles (EVs)to prevent extracellular degradation (104). A cell vesicle is a ubiquitous cellular carrier capable of transferring biomolecules and transmitting signals. Subcategories are effectively created when EVs have multiple functions, goods and compositions. The adaptive immune response is initiated by certain eukaryotic cells, such as B cells and dendritic cells, by presenting antigens through EVs. EVs are also used by other cell types, such as neurons, to improve action potentials and stimulate targets, and by adipocytes to process insulin in a paracrine manner. EVs can regulate cancer cell communication as well (105). The DNA, metabolites and proteins found in EVs secreted by normal cells can be used by tumour cells for’educating’non-cancerous cells to promote carcinogenesis through EVs. Using these vesicles, cancer cells can control the local environment and cellular processes, promoting inflammation and cancer development and evasion of the immune system.

In eukaryotes, EVs are enriched with certain classes of biomolecules, indicating the involvement of selective transport pathways. EVs can be used as novel biomarkers or therapeutic delivery systems for certain diseases and for the development of vaccines. EVs participate in various molecular pathways, including TLRs (106) (**Table 2**). LPS(TLR4), peptidoglycan (TLR2) and nucleic acids (TLR3/7/8/9)are a few biomolecules that interact with TLRs when attached to EVs. Many bacterial EVs bind to TLRs to promote disease or maintain homeostasis;however, the underlying mechanisms remain unclear. The IEC and *E. coli* C25 strains are symbiotic strains that interact *via* EVs. EV–IEC interactions upregulate TLR2/4/5 expression and promote IL-8 production, causing mild inflammation and preventing bacterial internalisation into the epithelium. The EVs of commensal *E. coli* cultures can induce the expression of TLR 1/5/6/7/8/9, IL-10, IL-12A and IL-1 in intestinal epithelial cells (107). Similarly, other commensal microbes interact with immune cells to affect gut homeostasis.

**Table 2:** Bacterial PRRs, PAMPs, and their corresponding signaling pathways.

PRR Classification	Type of PRR	PAMP	Signaling pathway
Toll-like receptor(TLR)	TLR1, TLR2	Triacyl lipopeptides, diacyl lipopeptides	NF-KB signaling
	TLR2,TLR6	Lipoteichoic acid, peptidoglycans, lipoproteins, pore proteins	NF-KB signaling
	TLR5	Flagellin	NF-KB signaling
	TLR6	Lipoteichoic acid, peptidoglycans	NF-KB signaling
	TLR9	Non-methylated CpG DNA	NF-KB signaling
	TLR4	Lipopolysaccharides	NF-KB signaling; TRIF signaling
Nucleotide-binding oligomerization domain-like receptor (NLR)	NOD1	Diaminopimelic acid	NF-KB signaling
	NOD2	Muramyl dipeptide	MAPK signaling; IRF signaling
	NLRP1	Muramyl dipeptide	NF-KB signaling
	NLRP3	RNA, DNA, muramyl dipeptide	NLR inflammasome
Stimulator of interferon genes (STING)	STING	Cytosolic DNA	NF-KB signaling; IRF3 signaling

PSA binds to TLR2 on DCs, thus enabling *B. fragilis* (NTBF)to promote intestinal immunotolerance. In the same fashion, *B. fragilis*(NTBF)-derived EVs are enriched with PSA, which activates TLR2-mediated increase in IL-10 production in DCs and enhances Treg production to protect against experimental colitis. *Bacteroides vulgatus*, an intestinal symbiont, releases EVs that activate TLR2 and TLR4 on CD11c^+^ DCs to promote anti-inflammatory responses (108). It is also possible for the health status of the host to influence how responsive the host cells are to commensal EVs. Specifically, EVs derived from *Bacteroides thetaiotaomicron*(Bt)stimulate the expression of IL-10 and IL-6 in DCs derived from healthy individuals, which in cell cultures from IBD patients, but EVs were ineffective, suggesting that TLR expression or components of the TLR pathway are altered. Therefore, EVs derived from commensal strains help to regulate homeostasis in the intestine through immunomodulatory antigens (109). However, interactions between pathogen-derived EVs and host cells result in the integration of microbial antigens within host cells, affecting intracellular processes and exacerbating the disease state (110).

IBD and cancer are exacerbated by EVs derived from pathogens (111). Innate immune responses are initiated by epithelial cells during immunosurveillance. *P. gingivalis*-derived EVs attract strong TLR2 (peptidoglycan)and TLR4 (LPS)responses and activate TLR7/8/9 (nucleic acids) (112) to a minor extent in the epithelium. *H. pylori*-derived EVs transfer virulence factors (i. e. CagA)that activate the TLR and NF-kB pathways in gastric and B cells, thus increasing the release of pro-inflammatory cytokines and cell proliferation in gastric neoplasm. *H. pylori* is a well-documented oncogenic bacterium that used EVs to initiate carcinogenesis. Other intestinal microbes may similarly use EVs to influence carcinogenesis. RNA within EVs derived from *Aggregatibacter actinomycetemcomitans* enhances the pro-inflammatory effects of TNF through TLR8 and NFkB (113). The delivery of DNA and RNA *via* EVs derived from *Staphylococcus aureus* can lead to potent IFN-β responses in macrophages *via* endosomal TLRs. EVs derived from the microbiome of rats with colitis can interact with TLR4 on IECs (114), thus triggering proinflammatory responses from macrophages, especially the release of IL-8. EVs in patients with IBD can induce pro-inflammatory responses, enhance the destruction of the intestinal epithelium and promote macrophage polarisation to M1 and M2 types (113). Peptidoglycan present in EVs can be internalised by IECs *via* TLR2 and transported to the basic membrane (115). After the peptidoglycan is transferred, EVs communicate with macrophages and induce the secretion of IL-6, which exacerbates inflammation in CRC. Therefore, EVs are very similar to their eukaryotic counterparts and represent an important component of intercellular interactions between microbes and host cells by interacting with TLRs (116).

### 6.2 EV-based immunotherapy for tumours

**Figure 5:**
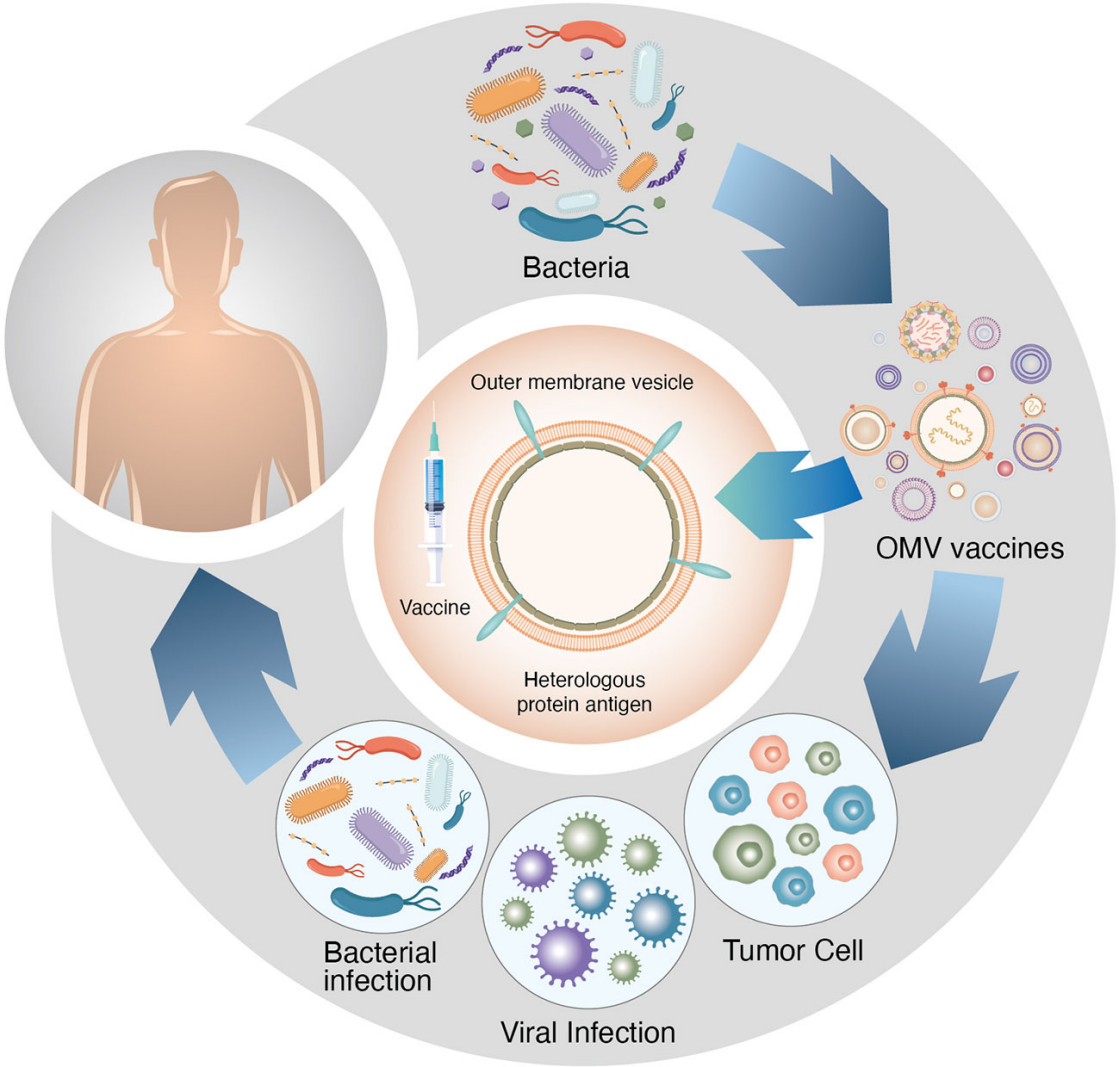
OMV vaccines play important roles in immunotherapy against various gastrointestinal tumours.

Cancer immunotherapy (**Figure 5**) has been performed using bacteria-derived vesicles. Owing to the abundance of PAMPs in bacteria, bacterial EVs are highly immunogenic and can attract and activate immune cells at tumour sites (117). However, intact bacteria pose safety concerns, and hence, bacterial ghosts whose intracellular contents have been removed are a safer alternative. Incorporation of adjuvants or antigens can enhance the efficacy and specificity of hollowed-out bacteria. Compared with their free-form counterparts, antigens incorporated into bacterial ghosts have demonstrated better retention, increased immune responses and improved tumour growth suppression after subcutaneous administration. When subcutaneously injected, EVs enable effective lymphatic drainage and enhance localisation to solid tumours through passive targeting. In addition to natural accumulation at tumour sites, EVs can be used therapeutically against multiple types of cancer because they can use local cancer cells as antigen sources in situ. For example, *E. coli*-derived EVs administered intravenously can eradicate CT26 and MC38 colorectal cancer, 4T1 metastatic breast cancer and B16BL6 metastatic melanoma (117).

Several lines of evidence support the potential capability of entrance of bacterial EVs into tumor microenvironment and modification of immune status. For example, Kim et al. showed that systematically administered bacterial outer membrane vesicles specifically target and accumulate in the tumor tissue, and subsequently induce the production of antitumor cytokines CXCL10 and interferon-γ. This antitumor effect is interferon-γ dependent, as interferon-γ-deficient mice could not induce such outer membrane vesicle-mediated immune response. This work showed remarkable capability of bacterial outer membrane vesicles to effectively induce long-term antitumor immune responses that can fully eradicate established tumors without notable adverse effects.

**Figure 6:**
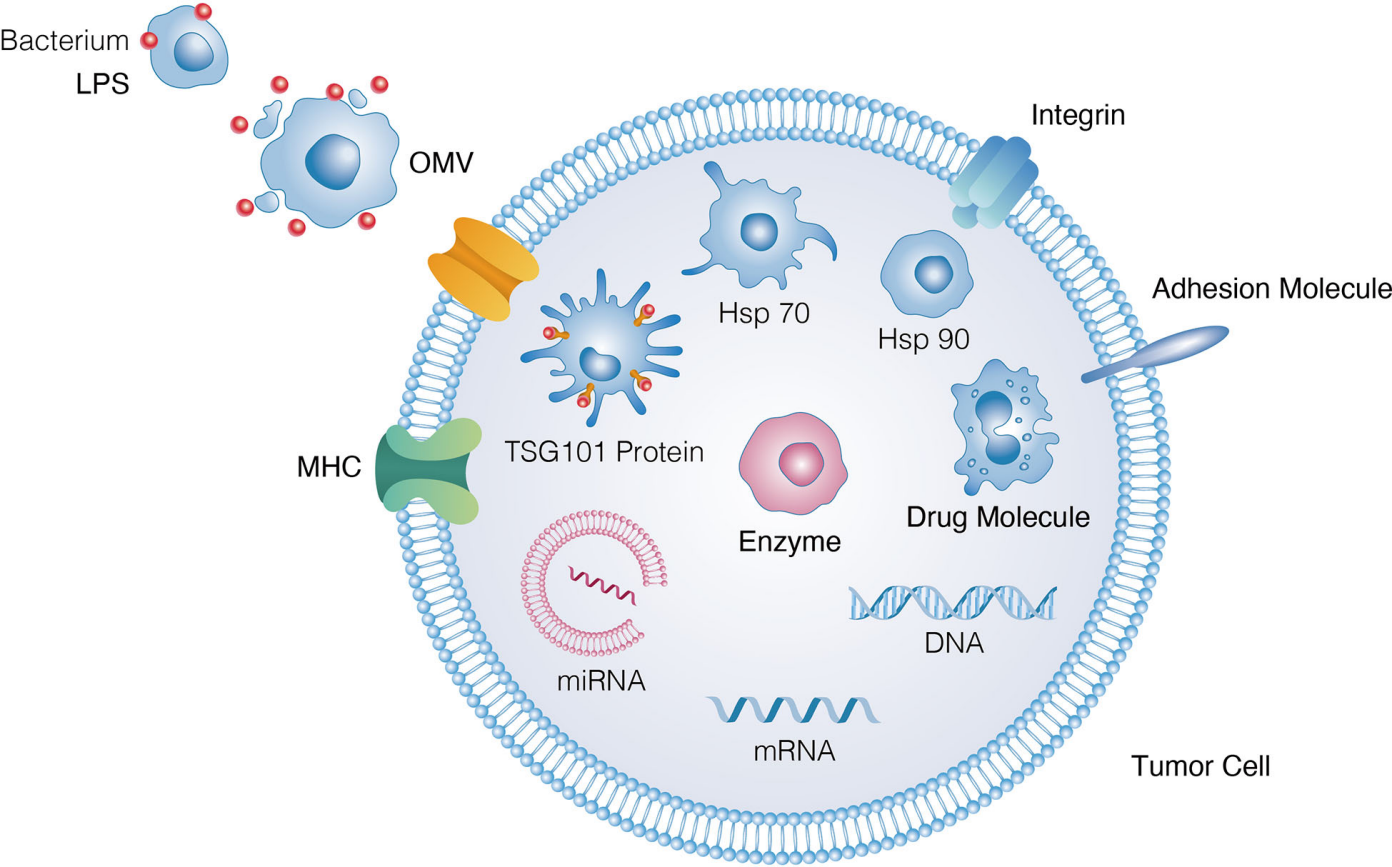
Biological effects that OMVs conferred to tumor cells and key molecules mediating such effects.

The combination of immunotherapy and EVs can synergistically enhance anti-tumour efficacy (**Figure 6**). Because EVs can localise to tumour sites, they can be encapsulated with chemotherapeutic drugs, including doxorubicin and paclitaxel (118, 119). Compared with the use of monotherapy, this combination therapy exhibited significantly greater inhibitory effects on tumour growth. In a study, EVs were coated with polymeric micelles containing tegafur, a fluorouracil prodrug derived from attenuated *Salmonella* that induces tumour cell apoptosis and sensitises cancer cells to cytotoxic T lymphocytes (120). Indocyanine green is another photosensitiser used in photoimmunotherapy. During laser ablation, a large amount of cancer antigens is released for immune priming after their accumulation in tumours (121). Through thrombosis and erythrocyte extravasation, *Salmonella typhimurium*-derived EVs sensitise tumours to photothermal therapy even in the absence of photothermal agents (59, 100, 122). To enhance the effectiveness of EVs, radiation therapy can also be used, which is considered a purely external method for destroying tumours. The efficacy of EVs can be enhanced using external tumour-destroying methods such as radiation therapy (123). This type of therapy has been used to eradicate B78 melanoma and NXS2 neuroblastoma in previous studies. Furthermore, loading of EVs onto micromotors is an innovative combination therapy, which causes considerable physical damage and destruction to tumours. Compared with non-propulsion micromotors, EV-loaded micromotors can effectively clear tumours at the primary site and improve the control of tumour growth distally in MC38, CT26 and B16-F10 tumour models (124).

## Implications

The purpose of this section is primarily to explore how insulin can be added to chemotherapy to treat gastrointestinal cancers, analyse and compare the effects of EVs and other methods combining EVs on gastrointestinal tumours and examine the novelty of EVs in treating gastrointestinal tumours in clinical settings.

Chemotherapy is an important treatment method for malignant digestive tract tumours. During chemotherapy, insulin treatment significantly reduces the effects of gastrointestinal tumours, and the efficacy of chemotherapy does not plunge (125). Insulin enhances the ability of tissues to absorb glucose and promotes anabolism in the liver, muscle and adipose tissues (125). In addition to improving metabolism and stimulating the growth and proliferation of normal and tumour cells, insulin can promote RNA and DNA synthesis in cells and tissues and induce tumour cell proliferation (126).

When insulin concentrations are within a certain range, it has no toxic effects on the human body. Studies have shown that insulin (2. 0–15. 0 mU/mL)can enhance the cytotoxic effects of etoposide on oesophageal and pancreatic cancer cells (127). In a study, large, medium and small doses of insulin combined with 5-Fu were found to be significantly more effective than 5-Fu alone in suppressing tumour growth in nude mouse S180 sarcoma and H22 liver cancer xenograft models (127). However, the number of white blood cells did not significantly differ between mice that received combination therapy and mice that receive monotherapy (P > 0. 05). Additionally, no difference was observed between the two groups of mice in terms of the morphology of the pancreas, liver and kidney. Therefore, combination therapy with insulin with chemotherapeutic drugs not only enhances the therapeutic efficacy but also reduces the toxicity of chemotherapeutic drugs (127). Using the abovementioned animal experiment as a basis, several clinical experiments were conducted by the authors. Chemotherapeutic regimens, such as DLF, are commonly used and highly effective for digestive system tumours. A study evaluated the efficacy of chemotherapy in preventing and treating hypoglycaemia;however, no significant differences were observed in chemotherapeutic efficacy between the experimental and control groups. The insulin group had larger or smaller lesions than the control group, depending on the size of the lesions, and the combination of insulin and chemotherapy was ineffective in the treatment of malignant tumours (125–127). In addition, it has an attenuation effect that does not reduce chemotherapy efficacy.

The combination of photothermal therapy and EVs has been successfully used to treat tumours of the digestive system. EVs are effective for specific anticancer therapy because they can target tumours (128). In a study, EVs elicited a photoacoustic signal in tumours immediately after intravenous injection, which increased during 120 minutes, indicating tumour accumulation. Several proteins and LPSs are present on the membrane surface of EVs, which may cause systemic toxicity. Studies involving immunocompetent mice have been conducted to address safety concerns regarding EVs. EVs induce significantly stronger TLR responses than EV and EV (100, 128, 129), suggesting that the latter two types of EVs were derived from a low-endotoxic *E. coli* strain that is generally well tolerated in a different way (100, 128). According to the results of studies on the use of bioengineered EVs in mice with cancer, the EV platform appears less likely to induce chronic systemic toxicity. The fate of EVs or melanin, which can function as a self-antigen, is an important question. Researchers have found that other EVs are degraded in late endosomes or lysosomes, and NADPH-dependent oxidoreductases degrade intracellular melanin. Because the bacterial membrane covers melanin, the immune system may be unaware of its presence in intact EVs (100, 128). As part of our future research, we will examine whether repeated administration of modified EVs in small and large animals results in systemic toxicity or toxic effects on major organs. In addition, it is important to explore synergies between photothermal therapy mediated by melanin and the immune responses triggered by EVs. Clinical applications require an evaluation of the long-term stability of EVs (100, 128). Previous studies have reported that EVs show clear melanin signals in both the skin and liver adjacent to the tumour. Therefore, the safety implications of EVs in healthy organs surrounding the tumour should be investigated. Photothermal therapy, in which light is irradiated on the tumour, may not necessarily cause toxic effects (100, 128).

An example of biomimetic nanoparticles(NPs)includes NPs fabricated using natural cellular materials for use in biomedicine (100, 128). In addition to retaining the original biological functions of the cellular material, biomimetic NPs can retain the physicochemical properties of synthetic nanoparticles. Consequently, NPs coated with cell membrane-derived components have attracted substantial attention in the biomedical field. This NP-based platform has multiple biomedical applications, especially cancer therapy with EV-coated NPs (100, 128). Biomedical applications of EVs, such as in photothermally active bacterial infections, have shown great promise. A few studies have reported the incorporation of NPs in biomimetic designs. It is encouraging that researchers are exploring non-conventional approaches to coating NPs with cancer cell membranes for biomedical applications. Cancer cell membranes have several unique properties that make them effective, including unlimited replication potential, immune tolerance, resistance to cell death, long circulation time and homologous binding capacity.

During chemotherapy, the most common side effects seen in patients with malignant digestive tract tumours are bone marrow suppression, nausea and vomiting, and more than 60% of patients cannot tolerate or complete the chemotherapeutic regimen as planned because of the high incidence rate of these side effects (130). Therefore, one of the current research directions in tumour treatment is to find a drug that does not reduce the efficacy of chemotherapy but reduces its toxic effects. Compared with immunotherapy, EV-based therapy has fewer side effects and more precise targeting. Cancer cells can be effectively destroyed by deciphering their DNA or RNA sequences at the genetic level instead of killing them superficially and disrupting normal metabolic functions (131).

## Conclusion

This review summarises the EVs of digestive tract microorganisms and their application in the treatment of digestive tract diseases. Several challenges limiting the use of EVs remain to be addressed: first, the safety and effectiveness of EVs should be improved;second, the structural integrity of EVs should be maintained; third, EVs should be homogeneous. To improve the safety and efficacy of EVs, genetic engineering can be used to modify them (132). The simultaneous expression of multiple antigens on EVs has a wide range of applications. The production of EVs can also be improved by engineering bacteria (122). Owing to the involvement of LPS in reducing the safety of EVs and inducing immune responses, it is extremely important to prepare EVs without LPS or with low levels of LPS. However, Pulido et al. immunised mice with *A. baumannii*-derived EVs in the presence or absence of LPS and found that EVs were 75% effective in mice without LPS, whereas the protection rate of EVs with LPS was up to 100%. Therefore, EVs containing LPS may have some protective properties. A gene knockout-based method was used by Lee et al. to prepare low-LPS *Salmonella typhimurium*-derived EVs (59, 100, 122). After these EVs were fused with OMPA, they substantially increased serum antibody levels in immunised mice. Sodium deoxycholate, a detergent, combined with EDTA is used to extract EVs with low LPS levels. Physical or chemical extraction of EVs may also selectively reduce LPS levels. EDTA- and proteinase K-treated EVs and intact EVs do not have significantly different effects on the expression of tumour-promoting factors, and responses to cancer may rely on the structural integrity of EVs. Nitrogen gas has been reported as a method for rapid preparation of EVs. EVs prepared using this method retain the integrity of bacterial membranes and are rich in membrane proteins. In addition to protecting mice against *Pseudomonas aeruginosa* infection, these EVs can improve their chances of survival. This nitrogen-based approach may become a novel method for vaccine preparation. EVs vary greatly among individuals. EVs derived from carbapenem-resistant *Klebsiella pneumoniae*(CRKP)can be modified to ensure homogeneity and improve immune efficacy (133). BN-EV-based vaccines with BSA NPs of controlled size can significantly increase the production of CRKP-specific antibody titres and improve the survival of mice (134).

Immunostimulatory properties of pathogen-associated molecular patterns are attractive for vaccine development;however, the potential risks associated with the introduction of bacterial toxins such as LPS into human patients should be neutralised. Toxic components in bacteria can be isolated more precisely from EVs by developing more efficient separation and purification techniques (99, 100, 135, 136). The development of novel genetic engineering approaches to reduce toxic factors is very promising, and these strategies may eventually lead to the production of EVs that are inherently safe and do not require complex handling. Additionally, repeated EV vaccinations may induce immune responses against the cellular carrier, which can decrease immunity to the intended target antigen (124). The density of the total antigen can be increased through various engineering approaches, and non-essential proteins that are particularly immunogenic can be removed or decreased in concentration. In the future, a multi-antigen drug should be rationally designed using EVs (100, 124). At present, clinically approved artificial serum preparations require the mixing of multiple antigens, which may simplify vaccine production.

Although there is significant room for improvement, great breakthroughs have been made in the development of EV-based platforms for the clinical management of microbial infections and gastrointestinal tumours (100, 124). Providing innovative solutions to address current challenges and consistent, researches on EVs will enable their widespread adoption primarily for clinical purposes.

## Author contributions

All authors listed have made a substantial, direct, and intellectual contribution to the work and approved it for publication.
